# Ultrafast spatiotemporal photocarrier dynamics near GaN surfaces studied by terahertz emission spectroscopy

**DOI:** 10.1038/s41598-020-71728-x

**Published:** 2020-09-03

**Authors:** Kota Yamahara, Abdul Mannan, Iwao Kawayama, Hidetoshi Nakanishi, Masayoshi Tonouchi

**Affiliations:** 1 Institute of Laser Engineering, Osaka University, Osaka, 565-0871 Japan; 2 Graduate School of Energy Science, Kyoto University, Kyoto, 606-8501 Japan; 3 SCREEN Holdings Co., Ltd, Kyoto, 612-8486 Japan

**Keywords:** Materials science, Optics and photonics

## Abstract

Gallium nitride (GaN) is a promising wide-bandgap semiconductor, and new characterization tools are needed to study its local crystallinity, carrier dynamics, and doping effects. Terahertz (THz) emission spectroscopy (TES) is an emerging experimental technique that can probe the ultrafast carrier dynamics in optically excited semiconductors. In this work, the carrier dynamics and THz emission mechanisms of GaN were examined in unintentionally doped n-type, Si-doped n-type, and Mg-doped p-type GaN films. The photocarriers excited near the surface travel from the excited-area in an ultrafast manner and generate THz radiation in accordance with the time derivative of the surge drift current. The polarity of the THz amplitude can be used to determine the majority carrier type in GaN films through a non-contact and non-destructive method. Unique THz emission excited by photon energies less than the bandgap was also observed in the p-type GaN film.

Gallium nitride (GaN) is one of the most important wide-bandgap semiconductors, which attracts a remarkable interest for light-emitting, high power, and high-frequency devices^1,2^. Despite great efforts, there are still many quality problems such as inefficient doping, defects, surface states, and defects in passivation, which occur in both crystals and films^3,4^. For instance, there is naturally a strong spontaneous polarization on the Ga face of GaN along the c-axis direction^5^. Due to this polarization and the defects in GaN, a typical AlGaN/GaN high-electron-mobility transistor (HEMT) functions as a normally-on (depletion mode) device or it must be operated with a back-gate^6,7^. There are still many challenges that need to be overcome to achieve better GaN devices and materials, and new material characterization tools are vital to advancing research in this field.

Terahertz (THz) emission spectroscopy (TES) and an imaging system known as a laser THz emission microscopy (LTEM) are emerging tools used to study the ultrafast dynamic carrier motion and displacement in optically excited materials^8,9^. Recently, we have demonstrated the application of LTEM on various semiconductors^10–12^. LTEM uses a femtosecond (fs) laser to excite the carriers in the materials. The carriers are accelerated by an electric field and they diffuse from the excited area, which induces a transient current, and THz waves are emitted in accordance with the time derivative of the photocurrent. The temporal THz waveforms reflect the ultrafast carrier dynamics, which are on the timescale of a few tens of femtoseconds^13^.

In wide-bandgap semiconductors, the penetration depth of light is shallow, and most photocarriers are generated near the semiconductor surface when the photon energy is larger than the semiconductor bandgap. Photoluminescence (PL) and electroluminescence can be used to characterize carrier recombination leading to photon emission for recombination times on the order of picoseconds to nanoseconds. Leitenstofer et al. has proven that, in GaAs, TES provides information on the instantaneous photo-response immediately after optical excitation^14,15^, whereas the conventional methods provide information on the carrier dynamics after carrier diffusion has occurred. With TES, information on mechanisms that occur on timescales less than a picosecond, such as carrier displacement speed and direction, can be obtained.

PL, photon absorption, and pump-probe reflectance measurements have been used to study the optical response of GaN, mainly by providing information regarding defects and carrier scattering. In the present work, TES was used to study the spatiotemporal optical responses of GaN. In general, THz emission from semiconductor surfaces is caused by the acceleration of photocarriers by a built-in field near the surface, as illustrated in Fig. 1, and/or photocarrier diffusion from the surface to the bulk. In the former case, electrons and holes drift in the opposite direction from each other, and in the latter case, diffusion is in the same direction but at different speeds, a phenomenon known as the Photo-Dember effect^13,16–19^, the model of which is often used to explain the THz emission mechanism from narrow bandgap semiconductors. Since the mobility of electrons in semiconductors is larger than the one of holes, thus, TES is expected to reveal such photoexcited electron dynamics, and the polarity of the THz wave amplitudes would indicate the majority carrier type in the GaN semiconductors. Here, we examined unintentionally doped n-type (#n-1), Si-doped n-type (#n-21), and Mg-doped p-type (#p-1) GaN films to obtain a clear understanding of THz emission from GaN. The THz emission waveforms from the samples under a variety of conditions were examined, and the THz emission mechanisms are discussed using the short-time approximation model from Ref^20^, together with the PL and Ultraviolet Visible Absorption Spectroscopy (UV–vis) measurements.

**Figure 1:**
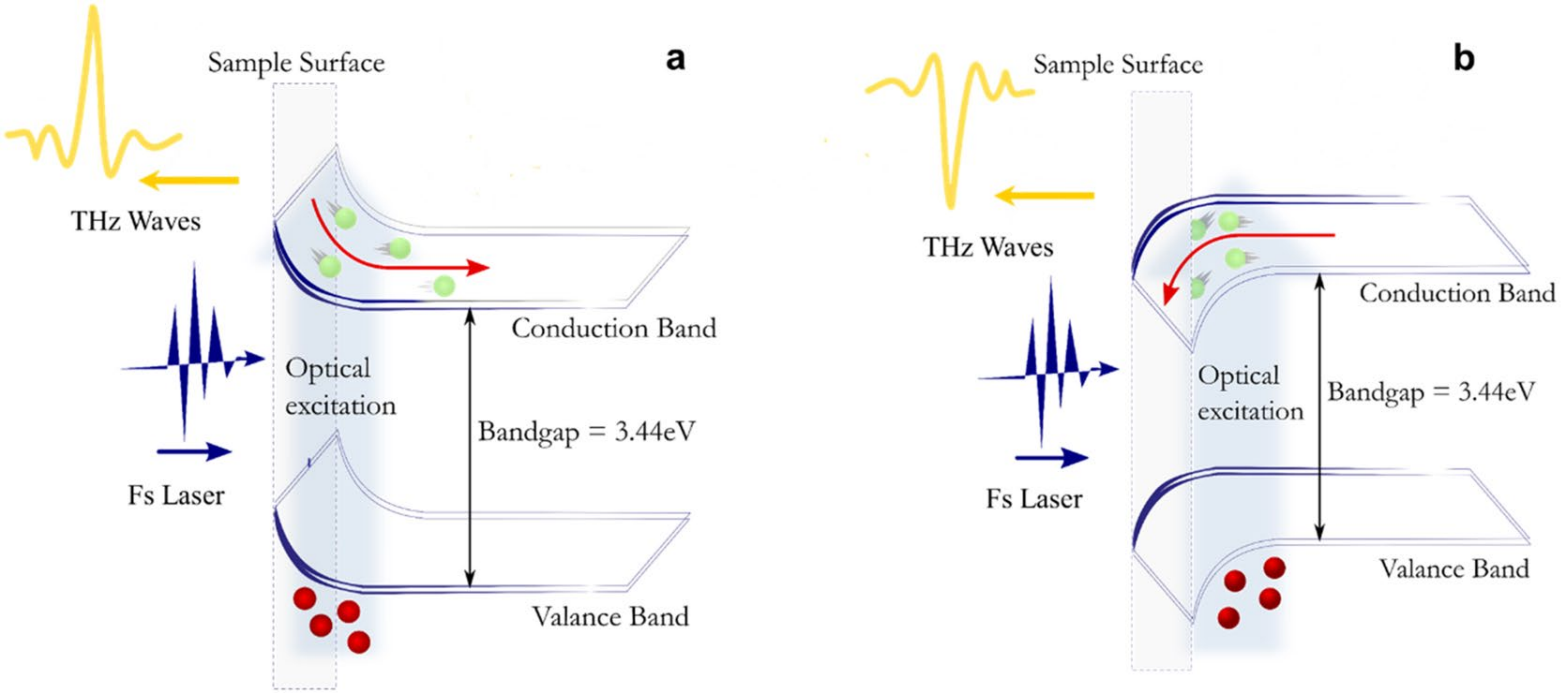
Schematic illustration of THz generation in **(a)** n-type and **(b)** p-type gallium nitride.

## Results

### Photoluminescence and UV–vis

**Figure 2:**
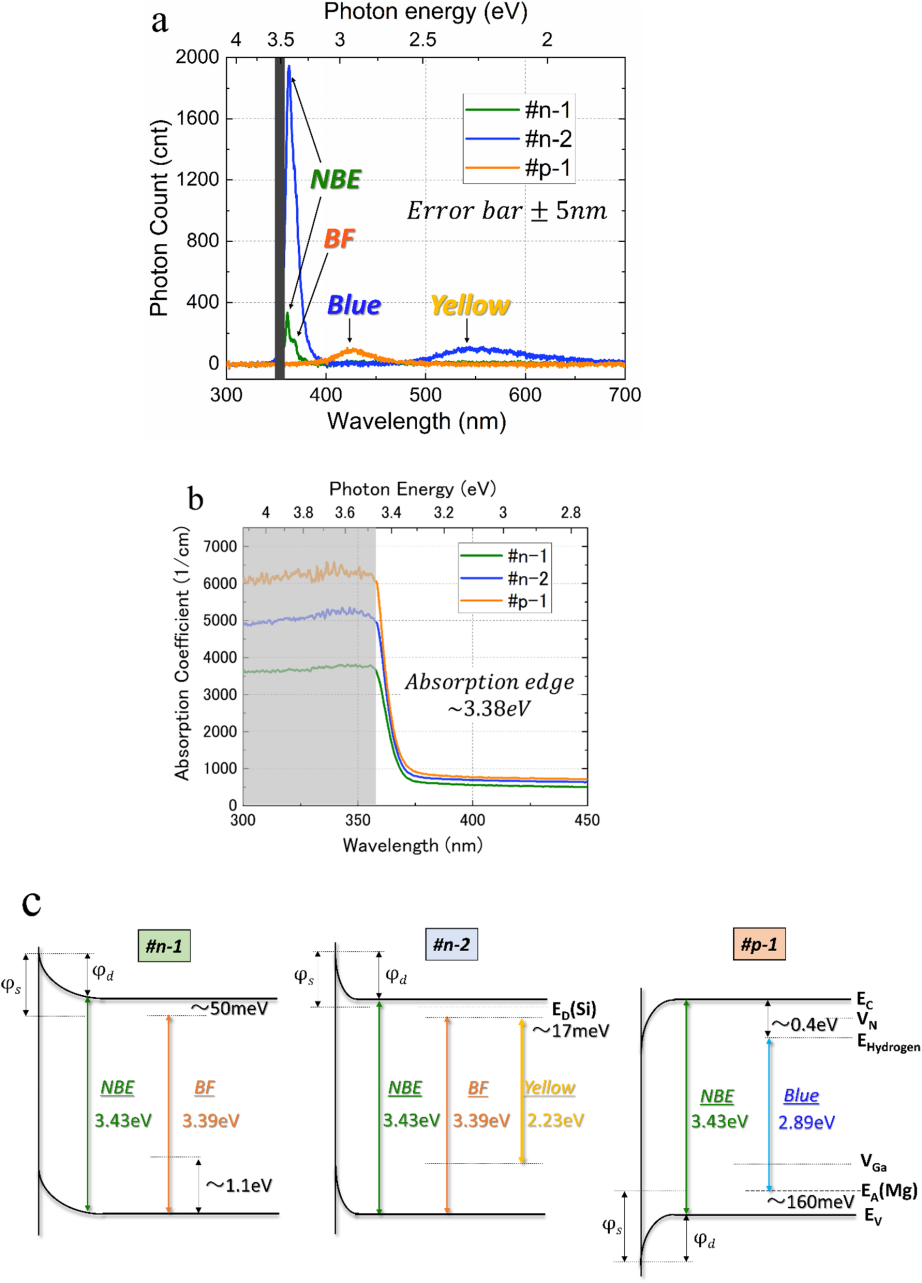
Optical response of the GaN film samples. **a** Photoluminescence spectra of each sample at an excitation wavelength of 356 nm. **b** Absorbance of each sample. **c** Energy band diagrams of each sample. Gray area is beyond the measurement limit where the penetration depths are shorter than the thickness of the samples.

Material descriptions for #n-1, #n-2, and #p-1in detail are given in the methods section. Figure 2a shows the PL spectra of the three GaN samples at room temperature (RT). The PL spectra of samples #n-1 and #n-2 show two dominant sharp peaks at 3.43 and 3.39 eV. The peak at 3.43 eV corresponds to the near-band-edge (NBE) emission of GaN. The peak at 3.39 eV is considered a bound-free transition (BF) due to the recombination of holes in the valence band with electrons from donor levels or nitrogen vacancies. Evwaraye et al. reported that the activation energy of a shallow donor is approximately 50 meV, which correlates with the nitrogen vacancies (V_N_) in undoped GaN^21^. Since Gotz et al. determined the activation energy for the ionization of Si donors in GaN to be 12–17 meV using variable temperature Hall effect measurements^22^ the Si donor level should be apparent in the spectra of sample #n-2, but it is not. The broad emission centered at 2.23 eV is attributed to emission via recombination between carriers from N and Ga vacancies, which leads to yellow luminescence. J. Neugebauer et al. calculated the Ga vacancy (V_Ga_) level which is responsible for the acceptors to capture the photoexcited carriers to emit the yellow luminescence (See Fig. 2c.) to be 1.1 eV above the top of the valence band^23^. Sample #p-1 exhibits a dominant broad peak centered at 2.89 eV, which is due to the recombination occurring between the donor and acceptor levels of hydrogen leading to blue luminescence^24^. The activation energy (E_A_) of Mg-acceptors was measured to be 160 meV by P.Kozodoy^25^ and M. A. Reshchikov et al. reported that deep donor energy levels that are approximately 0.4 eV below the conduction-band minimum may be due to defects related to the hydrogen in Mg-doped GaN grown by MOCVD^c26^. The energy difference between the Mg-acceptors and hydrogen defects coincides with the blue luminescence.

The absorbance spectra of the GaN film samples are shown in Fig. 2b, revealing that sharp absorbance starts at around a wavelength of 360 nm. From the absorption data, the band edge was estimated to be about 3.38 eV for all the samples. This value is almost the same as the previously reported value of 3.39 eV^27^. An absorption band edge of 3.38 eV corresponds with the excitation transition energy from the valence band to the nitrogen vacancies. The absorbance of the doped samples (#n-2 and #p-1) are higher than that of the non-doped (#n-1) sample, which is attributed to the photon absorption by the free carriers^28^. Based on the absorbance measurements, the penetration depths for samples #n-1, #n-2, and #p-1 are estimated to be 1.07, 0.78, and 0.65 μm, respectively. ^29^.

Based on the above measurements, and data from references, the GaN samples are expected to have the energy band structures shown in Fig. 2c.

### Terahertz emission properties

**Figure 3:**
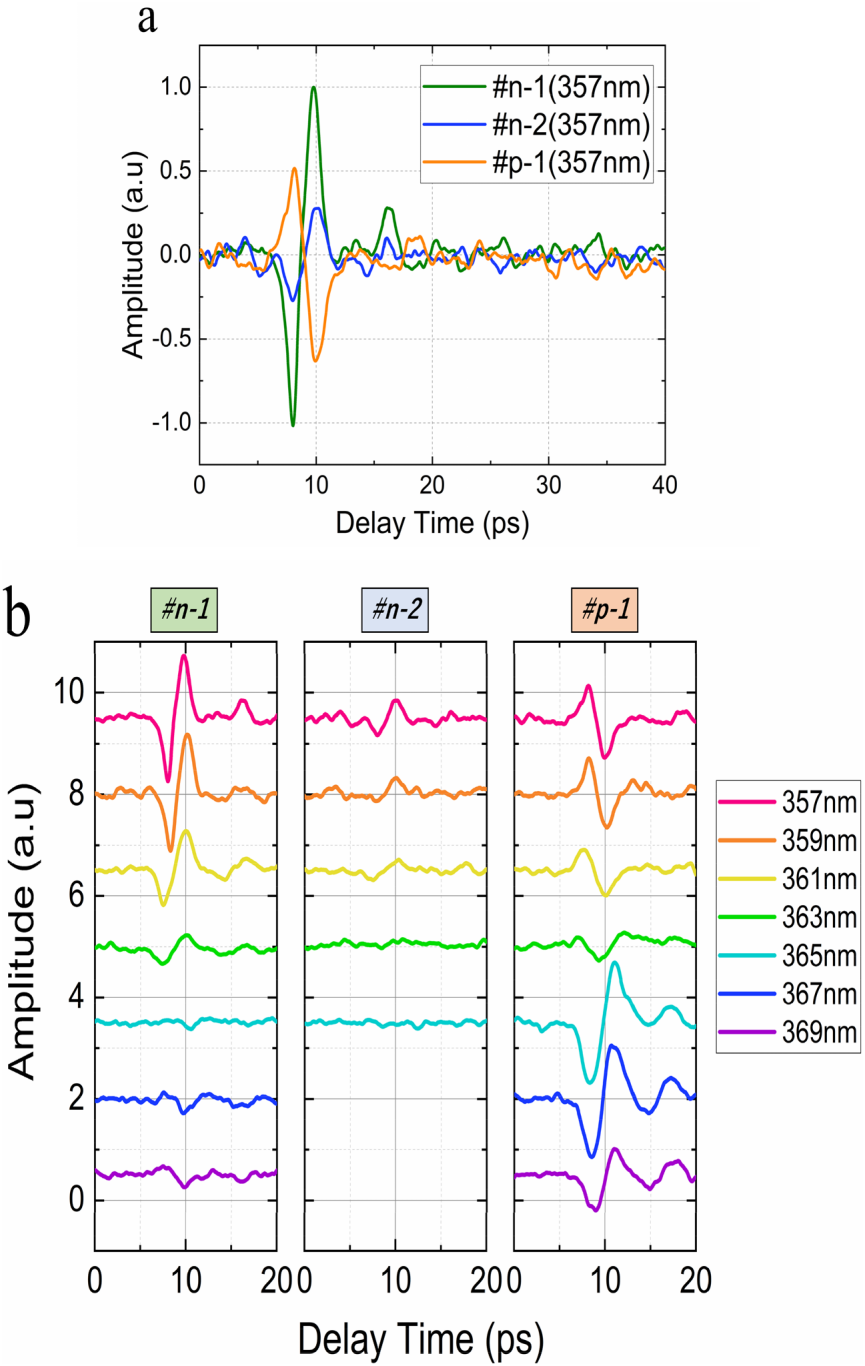
THz emission of the GaN film samples. **a** Waveforms of each GaN sample with an excitation wavelength of 357 nm. **b** Waveforms of the THz radiation from the samples at different excitation wavelengths.

The terahertz emission waveforms of the three GaN film samples are shown in Fig. 3a. The excitation wavelength and pump power used were 357 nm and 15 mW, respectively. The wavelength corresponds to a photon energy ${E}_{p}$ of 3.47 eV, which is larger than the bandgap energy of GaN (3.43 eV), and it is sufficient to excite an electron from the valence band to the conduction band. The amplitude at 10 ps for sample #n-1 is higher than that of sample #n-2, and their amplitude sign is opposite to that for sample #p-1. We used InAs semiconductor as a reference THz emitter. It is well known that THz emission from InAs is based on Photo-Dember effect and during optical excitation the diffusion current direction is from bulk towards the surface. Therefore, the positive peak of THz emission from n-type samples, corresponds the current flows from bulk to surface, whereas the negative peak of THz emission from p-type sample, corresponds to photocurrent flows from the surface to the bulk in the sample.

The THz emission properties is expected to be strong dependent on the excitation wavelength when the photon energy changes from smaller to larger than the bandgap. Figure 3b shows the THz emission waveforms from the three samples for various excitation wavelengths at a constant pump power of 15mW. As expected for samples #n-1 and #n-2, below the excitation wavelength of 365 nm, the waveform amplitude decreases with increasing wavelengths, until the waveform disappears above an excitation wavelength of 365 nm, which corresponds to photon energies less than the bandgap. Sample #p-1 shows a similar behavior above the bandgap excitation energy except with opposite amplitude polarity. However, below the bandgap excitation energy, unexpected THz emission is observed. Even at wavelengths below 365 nm, strong emission is detected, and the amplitude polarity changes. The results imply that free carriers are excited in sample #p-1 even for photon energies less than the bandgap, and the electrons diffuse inward from the film surface. For sample #p-1, it is possible that free carriers were excited from the doped Mg energy states to the conduction band and/or from the valence band to the degenerate conduction band for the enlarged penetration depth of the photons, which may allow us to neglect the carrier drift due to the built-in field near the surface. Note that PL emission from NBE and EF were not observed in sample #p-1.

**Figure 4:**
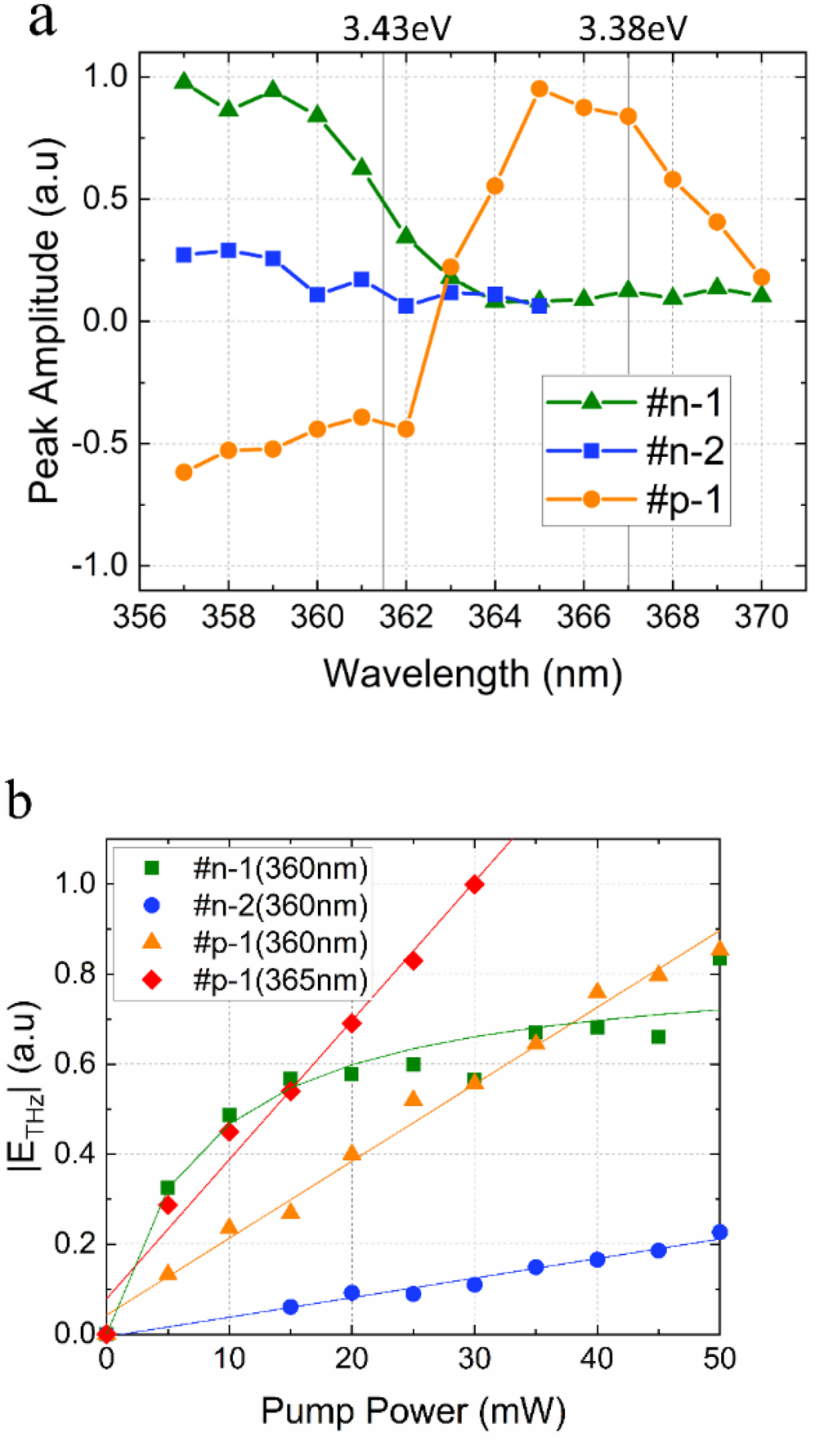
**a** The peak THz amplitude around t = 10 ps of the waveforms from Fig. 3b as a function of excitation wavelengths. **b** The terahertz intensity dependence on laser pump power.

Figure 4a shows the peak amplitude of the THz emission at approximately 10 ps as a function of excitation wavelength. From approximately 363 nm, the THz emission intensity increases with decreasing excitation wavelengths for all the samples, but the polarities for the n and p-type samples are opposite to each other. The emission amplitude of sample #p-1 crosses the zero line from negative to positive values above 363 nm and a maximum intensity was observed at approximately 366 nm, which is close to the energy of nitrogen vacancy states. However, as we will discuss in the following session, this behavior is attributed to the shift from the drift to the diffusion model.

The power dependence of the waveform’s peak intensity at excitation wavelengths of 360 nm for all the samples and of 365 nm for sample #p-1 was shown in Fig. 4b. At low laser power fluencies, the THz emission amplitude from the semiconductor surface is expected to increase in proportion to the excitation power and the THz emission amplitude of sample #n-1 saturates with increasing laser power. The saturation behavior is generally due to the screening of the built-in field by electron–hole displacement. The sample #n-1 has a larger electron mobility, which would cause this screening effect at low laser powers. These features are commonly observed in conventional semiconductors.

The azimuthal dependence of THz emission was also examined to determine if the THz generation mechanism is based on nonlinear effects caused by the second-order susceptibility χ^(2)^. We observed no clear azimuthal dependence and concluded that the emission properties do not include nonlinear generation effects. The details of the azimuthal dependence study are presented in the supplementary information.

## Discussion

As the above results have shown, the THz emission properties include a variety of carrier dynamics. The main interest of the present work is to obtain an intuitive understanding of the THz emission mechanism for various types of GaN. Here, the THz emission mechanism is discussed using an ultrafast time-domain approximation model. The timescale of interest is less than 500 fs, which is shorter than ensemble dynamics such as carrier diffusion. Within this timescale, THz emission is governed by the photocarrier acceleration due to the built-in field or diffusion just after excitation. Important parameters to consider when describing THz emission are the surface potential, the density of carrier doping, the width of the depletion layer (*w*), and carrier mobility. *w* in n-type semiconductors is estimated using the following equation.

1 $$ w = \sqrt {\frac{{2\varepsilon_{r} \varepsilon_{0} V_{D} }}{{e^{2} N_{D} }},} $$

where *ε*_*r*_ is the dielectric constant (*ε*_*r*_ = 9.5^30^. ), *ε*_*0*_ is the vacuum permittivity, *V*_*D*_ is the diffusion potential, *e* is the electron charge, and *N*_*D*_ is the donor concentration. It is often difficult to obtain doping density information, and so, it was approximated using the measured carrier density *n*. In the present case, the thickness of the depletion layers is estimated to be 112.7, 37.0, and 42.8 nm for samples #n-1, #n-2, and #p-1, respectively. The penetration depths λ_L_ were determined from the absorbance measurements. The estimated parameters are summarized in Table 1.

**Table 1:** Summary of calculation results from each measurement, which are correlated with THz radiation. E_Max_ is the built-in field at the surfaces.

Samples	W (nm)	V_D_ (eV)	E_Max_ (Mv/cm)	λ_L_ (3.45 eV) (μm)	μ_e_|V_D_| / λ_L_ (a.u.)
#n-1	112.7	1.21	0.11	1.07	340
#n-2	37.0	1.30	0.35	0.78	284
#p-1	48.2	-1.42	0.29	0.65	302

Excluding the THz emission mechanism by nonlinear generation, the major mechanisms are by the drift and/or diffusion photocurrent. In the ultrafast approximation model^20^, the photocarrier acceleration field is assumed to be *E*_*Max*_. For *w* < *λ*_*L*_ (penetration depth), the amplitude of the THz electric field (*E*_*THz*_*)* generated by a drift photocurrent is written as

2 $${E}_{THz}\propto {\mu }_{e}{E}_{Max}{I}_{P}\propto {\pm \mu }_{e}\frac{{V}_{D}}{{\lambda }_{L}}{I}_{P}$$

where *μ*_*e*_ is the electron mobility and *I*_*P*_ is the injection photon number. The sign of *E*_*THz*_ changes in accordance with the sign of *V*_*D*_. The values of ${\mu }_{e}\frac{\left|{V}_{D}\right|}{{\lambda }_{L}}$ were calculated to be 340, 284, and 302, which qualitatively agrees with the observed amplitudes. Since the photocurrent due to the electron diffusion inward the bulk should show no sign change of the THz amplitude, which support that the THz emission from the GaN surface originates in the drift photocurrent generation.

**Figure 5:**
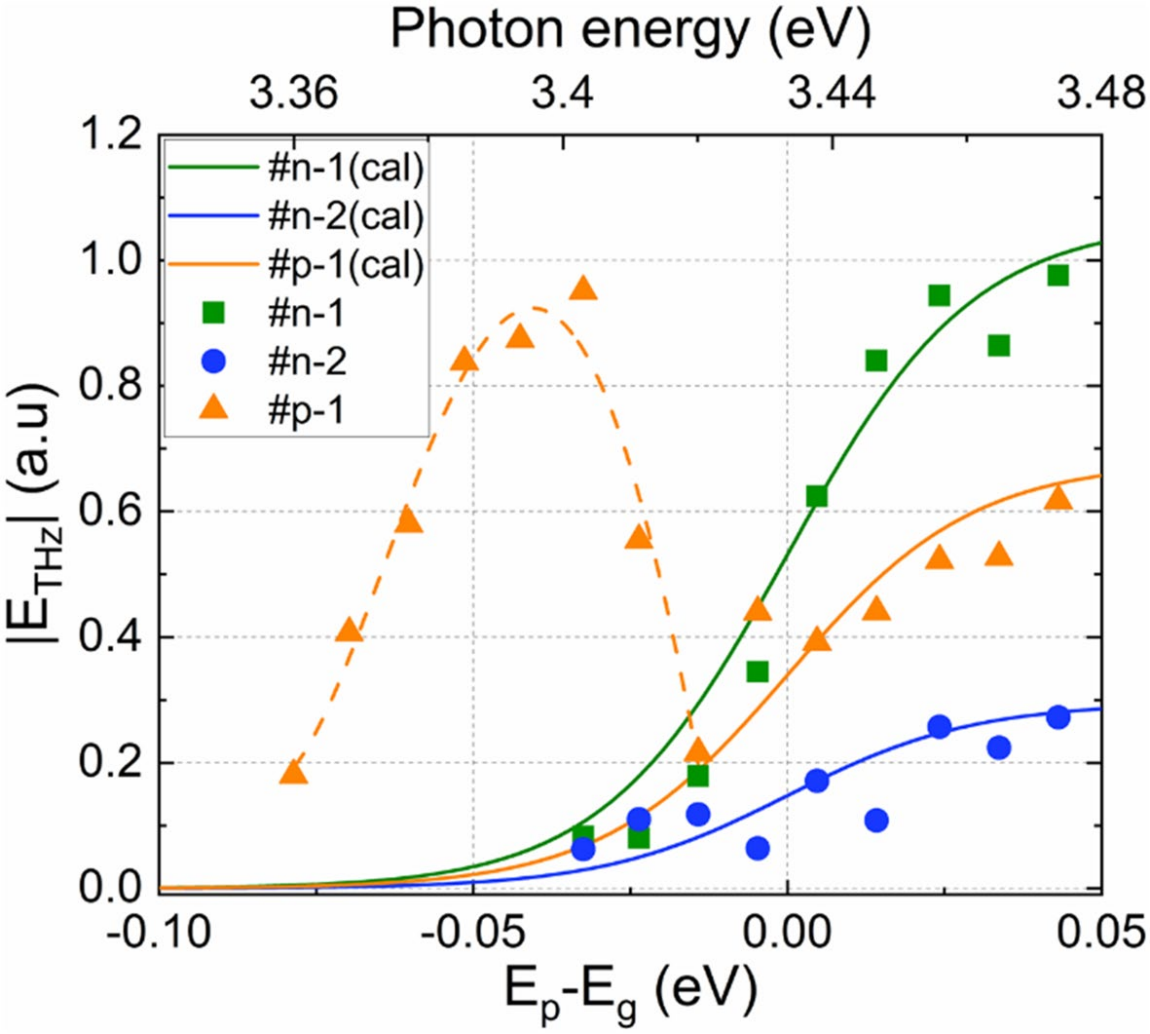
Relationship between excess photon energy (Ep-Eg) and terahertz intensities. The solid lines are the calculated fits to the intensities with the calibrated photon numbers.

For the excitation close to the conduction band edges, we can assume the parabolic relationship between the electron wave vectors and energies, resulting the mobility constant. Then Eq. (2) suggests that the emission amplitude is independent of the excess photon energy (i.e. the difference between the photon energy and the semiconductor bandgap, E_p_-E_g_). Figure 5 shows how the maximum THz radiation intensities change with excess photon energy. The intensities increase with increasing excess energy for all the samples. To explain this, we considered the excited carrier density excited with the fs optical pulses. Due to the wavelength broadening of the fs laser electric field, the carrier density should be calibrated. Assume that the fs pulses have hyperbolic secant distribution, the emission amplitude can be described by,

3 $$\left|{E}_{THz}\right|={\int }_{0}^{{E}_{p}}A\cdot {sech}^{2}\left[B\left(E-{E}_{g}\right)\right]dE,$$

where $A$ and $B$ are fitting parameters. In the figure, the solid lines are the fits to the THz intensities with $A$ of 18, 5, 11.5, for the samples #n-1 #n-2, and #p-1, respectively, $B$ of 0.02954, and a wavelength width of ± 2.7 nm^31^. This clearly explains that the increase in the intensity is due to the excited carrier density. This implies that the THz emission amplitude has no significant dependence on the excess photon energy. Thus, it can be concluded that when the excitation powers are larger than the bandgap energy, then THz emission is caused by photocarrier drift accelerated by the built-in field.

The THz excitation at energies below the bandgap that was observed for sample #p-1 is also discussed here. In Fig. 5, the orange dotted line is a guide to the eye. It shows that the maximum intensity is emitted at wavelengths around 366 nm, which corresponds to an energy of approximately 40 meV below the conduction band. This energy corresponds to N vacancies. Based on the band diagram for sample #p-1 in Fig. 1, the excitation from the valence band to the N vacancies could be the origin of the absorption and THz excitation. The conduction band edge is smeared and forms a degenerate band rather than isolated defect states. However, the emission is also proportional to the mobility, as suggested by Eq. (2), which would be much smaller in the degenerate band than in the conduction band. It is speculated that a narrow degenerate band near the conduction band edges decreases the bandgap slightly, and a long penetration depth induces the THz emission by the carrier diffusion facilitated with the thermal phonon scattering, and the THz excitation mode changes from diffusion in the band to the drift model with increasing excitation energy, when the penetration depth is shortened. A small shift of the positive amplitude to the longer time region is seen in Fig. 2b which suggests a decrease in mobility. Or there might exist a large number of acceptor-type defects near the valence band. Further discussion requires different experimental measurement approaches such as pump and probe THz emission spectroscopy^32^, and techniques that measure temperature dependence.

In summary the optically excited carrier dynamics of unintentionally doped n-type, Si-doped n-type, and Mg-doped p-type GaN films were studied using THz emission spectroscopy. The photons excite the photocarriers near the surface, which drift inward and outward due to the built-in fields and cause the emission of THz radiation into the free space. THz emission that results from excitation by photons with energies greater than the bandgap is attributed to a surge drift current rather than a diffusion current caused by the Photo-Dember effect, although the depletion layer in GaN is ultrathin compared to the optical penetration depth. It was confirmed that the polarity of the amplitude reveals the majority carrier type in the GaN films. For the Mg-doped p-type GaN film, unique THz excitation by photons with energies less than the bandgap was observed, which is attributed to photocarrier diffusion in a thin degenerate band near the conduction band edge that was formed by N vacancies and/or hydrogens. The results prove that TES and LTEM have a strong potential as nondestructive/noncontact tools for semiconductor research and development, especially for wide-bandgap semiconductors.

## Methods

### Material description

Three types of GaN single-layer film samples were grown using metal–organic chemical vapor deposition (MOCVD) on c-plane sapphire (0001) substrates (Suzhou Nanowin Science & Technology Co. Ltd.) at a thickness of 4.5 ± 0.5 μm. The sample information is given in Table 2. The carrier density and carrier type are quoted from the manufacturer’s datasheet, the mobility was calculated based on electron beam induced current (EBIC) measurements, and the Ga-surface potential was estimated from KFM measurements using the Sze and Cowley model (χ_GaN_ = 3.5 eV)^33–37^. The crystalline structure is a wurtzite crystal with a- and c-axis lattice constants of 3.19 Å and 5.19 Å, respectively.

**Table 2:** Specifications of the different GaN film samples.

Samples	Dopant	Carrier type	Carrier density (cm^-3^.)	Electron mobility μ_e_ (cm^2^./Vs)	Surface band-bending φs (eV)
#n-1	Undoped	n-type	< 1 × 10^17^.	300	1.26^b^.
#n-2	Si	n-type	> 1 × 10^18^.	170^a^.	1.32^c^.
#p-1	Mg	p-type	6.41 × 10^17^.	139^a^.	-1.58^c^.

^a^.Ref^38^.

^b^.Ref^39^.

^c^.Ref^40^.

### Photoluminescent spectroscopy and UV–VIS measurements

The PL and optical absorption were measured with a fiber optic spectrometer (HORIBA iHR320) and UV–vis spectrophotometer (HITACHI U-4100). A femtosecond laser was used for PL measurements and the THz measurements. The excitation wavelength was centered at 356 nm. There is some uncertainty in the wavelength since the fs laser was used as the excitation source.

### THz emission spectroscopy

**Figure 6:**
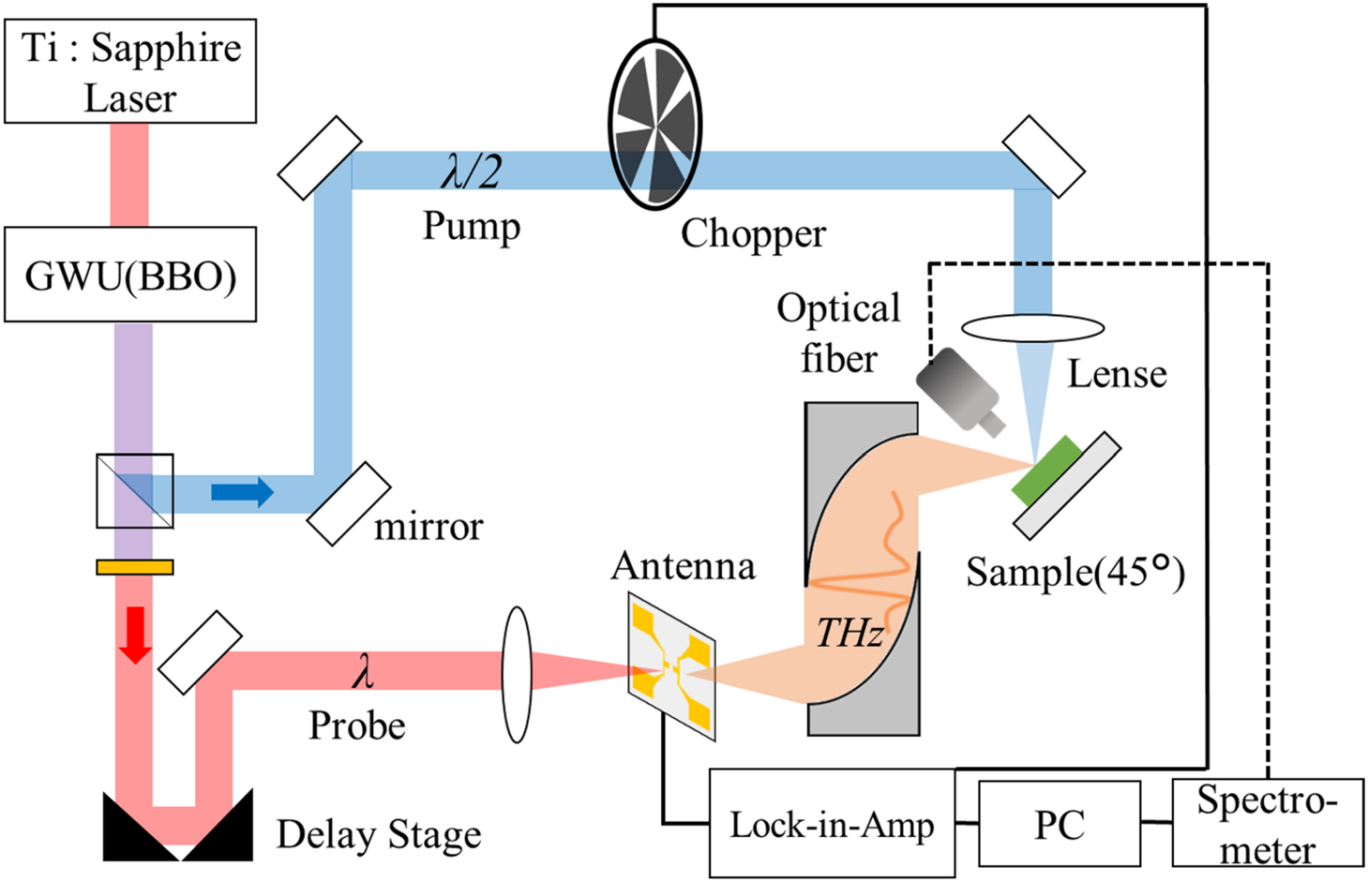
Schematic diagrams of the LTEM system.

A schematic of the THz emission spectroscopy system is shown in Fig. 6. The THz emission spectroscopy system utilizes a Ti:sapphire laser with a center wavelength of 714–740 nm, a pulse width of approximately 100 fs, and a repetition rate of 80 MHz (Spectra-Physics MaiTai HPTK-W) as the excitation source. In order to excite GaN, we used a wavelength converter (Spectra-Physics GWU-23FSHT-W) for the second-harmonic generation with a barium borate (BBO) crystal. A pump light (blue line) was modulated using an optical chopper and focused on the sample with a spot diameter of approximately 100 μm. A probe light (red line) was used to generate photocarriers in a dipole-type photoconductive antenna (PCA) that was made of low-temperature grown GaAs, and a power of 10 mW was used to detect the THz waves in the time domain. The details of the THz time-domain emission spectroscopy system have been reported elsewhere^5,41^.

## Supplementary information

Supplementary file1

## Data Availability

The data that support the findings of this study are available from the corresponding author upon reasonable request.
