# Supplementary material for: Ultrafast spatiotemporal photocarrier dynamics near GaN surfaces studied by terahertz emission spectroscopy
Source: Sci Rep. 2020 Sep 3;10:14633. doi: 10.1038/s41598-020-71728-x (PMC7471959; doi:10.1038/s41598-020-71728-x)
Supplement: Supplementary file 1 — Supplementary file1 [file 41598_2020_71728_MOESM1_ESM.pdf]

Supplementary Information

Ultrafast Spatiotemporal Photocarrier Dynamics near GaN Surfaces Studied by Terahertz Emission Spectroscopy

*Kota Yamahara[1], Abdul Mannan[1], Iwao Kawayama[1,2], Hidetoshi Nakanishi[3], Masayoshi Tonouchi[1]**

[1] Institute of Laser Engineering, Osaka University, Osaka 565-0871, Japan
[2] Graduate School of Energy Science, Kyoto University, Kyoto 606-8501, Japan,
[3] SCREEN Holdings Co., Ltd.,Kyoto 612-8486, Japan

E-mail: tonouchi@ile.osaka-u.ac.jp

In-plane direction dependence. Figure S1 shows the relationship between the c-axis of the GaN sample, the emitted THz wave, and the fs excitation laser. The excitation laser had an incident angle of 45°. The terahertz emission waves from sample #n-1 rotated by 0 to 330° in the plane are shown in Fig S2a). The excitation wavelength and pump power used are 360 nm and 10 mW, respectively. The amplitude peaks at 10 ps and the THz waveform shape is almost the same for all the angles. The maximum intensity of the terahertz wave was constant at approximately 0.8 a.u., as shown in Fig.S2b), and there was no significant change in the THz wave intensity in the c-plane. This insensitivity to the angle is because of the uniform polarization directions of the c-plane and the minimal deviations and defects in the film thickness. As there is no in-plane directional dependence, the THz radiation is not due to nonlinear optical effects.

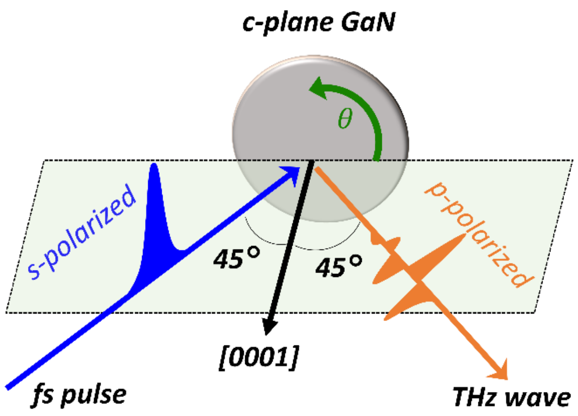

Fig. S1 Schematic diagram of the crystal axes of c-plane UID-GaN and the propagation directions of the fs laser and THz wave.

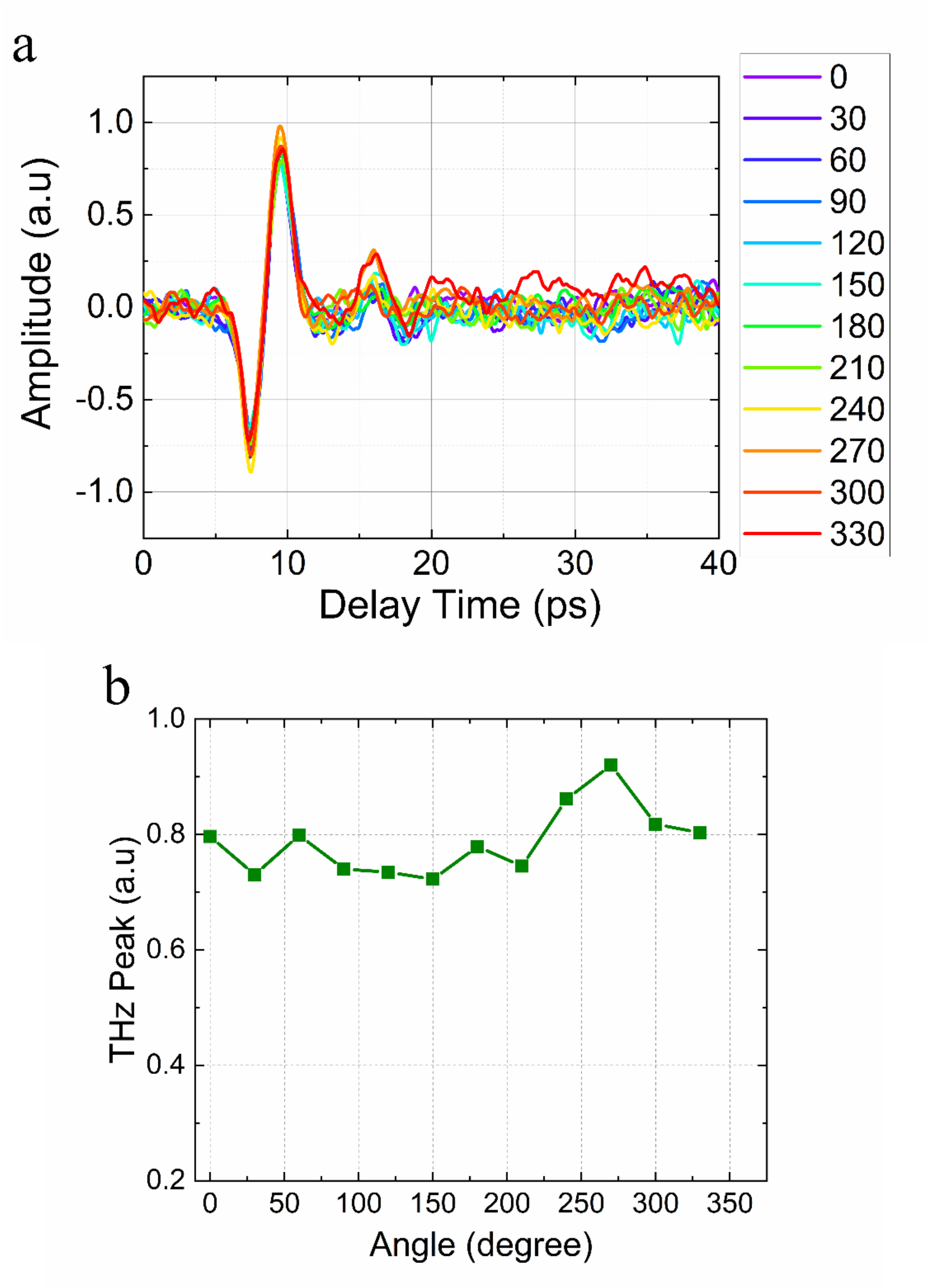

Fig. S2 Angle dependence of sample #n-1 in the c-plane (a) Rotational in-plane angle dependence of terahertz radiation waveform from sample #n-1 with an excitation wavelength of 360 nm and laser power of 10 mW. (b) Rotational in-plane angle dependence of the THz peak amplitude.
